# Changing the culture: impact of a diagnostic stewardship intervention for urine culture testing and CAUTI prevention in an urban safety-net community hospital

**DOI:** 10.1017/ash.2024.12

**Published:** 2024-01-29

**Authors:** Alfredo J. Mena Lora, Jessica Hua, Mirza Ali, Candice Krill, Eden Takhsh, Susan C. Bleasdale

**Affiliations:** 1 University of Illinois at Chicago, Chicago, IL, USA; 2 Saint Anthony Hospital, Chicago, IL, USA

## Abstract

Cultures from urinary catheters are often ordered without indication, leading to possible misdiagnosis of catheter-associated urinary tract infections (CAUTI), increasing antimicrobial use, and *C difficile*. We implemented a diagnostic stewardship intervention for urine cultures from catheters in a community hospital that led to a reduction in cultures and CAUTIs.

## Background

In the United States, 12%–16% of adult inpatients will have a urinary catheter during their hospitalization and approximately 50% will have a catheter without a valid indication.^1,2^ Urinary catheters can lead to inflammation and colonization of the urinary tract, compromising the accuracy of tests such as urinalysis and urine cultures. Due to these limitations, the Society of Critical Care Medicine (SCCM) and the Infectious Diseases Society of America (IDSA) advise against the routine use of urine cultures for the workup of fever in critically ill patients with catheters unless symptoms are present, highlighting the importance of a thorough history and physical examination to guide possible diagnostic studies for infectious and noninfectious sources.^3,4^ These guidelines recommend urine cultures for the workup of fever in transplant recipients, neutropenia, genitourinary surgery, and obstruction.^3,4^ However, urine cultures are frequently ordered without indication.^1,2^ Indiscriminate testing can lead to misinterpretation of results, erroneous diagnosis of catheter-associated urinary tract infections (CAUTIs), excess antimicrobial use, resistance, and *C difficile* infections.^1^ CAUTI misdiagnosis can increase CAUTI rates and have an impact on hospital quality measures and reimbursement.

Diagnostic stewardship can improve evidence-based testing and mitigate over-testing.^1^ Studies in critical care settings and large hospitals have shown a reduction in CAUTI rates.^5,6^ However, there is a paucity of data in smaller community hospitals. We deployed a hospital-wide urine culture stewardship initiative at a 151-bed urban safety-net community hospital to evaluate its impact on urine culture ordering practices and CAUTI rates.

## Methods

### Study design

**Figure 1.**
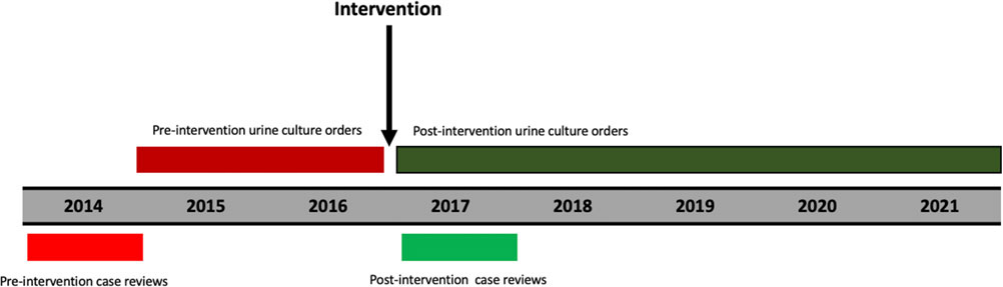
Timeline of intervention, case reviews, and urine culture orders.

We performed a quasi-experimental study incorporating diagnostic stewardship for urine cultures from urinary catheters. The intervention started in January 2017 as part of a comprehensive CAUTI reduction program (Fig. 1).^7^ A memorandum of evidence-based recommendations for urine cultures from urinary catheters was distributed to the medical and nursing staff and posted in medical units (Supplement A). Nurses were instructed to contact the infectious diseases (ID) physician when cultures from urinary catheters were ordered (Supplement B). Cases were prospectively audited and a holistic evaluation was performed by the ID physician before sending the sample to the laboratory. Feedback was provided to the ordering provider if indications were not met, and the urine culture canceled if the ordering provider agreed.

Data on each case was collected prospectively for twelve months, including case description, duration of indwelling urinary catheterization, urine cultures ordered per patient, and cause of fever (Fig. 1). Cases reviewed by the hospital CAUTI surveillance program from 2014 were compared with the first-year post-intervention. This year was selected due to lack of CAUTIs in years immediately prior to the intervention because of a comprehensive CAUTI reduction program.^7^

A report was generated with urine cultures ordered for hospitalized patients, including those with and without urinary catheters. Rate of urine cultures per 1,000 patient days for patients with urinary catheters, percent of urine cultures collected from urinary catheters, and rate of CAUTIs per 1,000 patient days were calculated one year before and 5 years after the intervention. Due to electronic medical record (EMR) implementation, data on urine culture orders was unavailable prior to 2015.

### Data analysis

Descriptive statistics were used to summarize data. The pre-intervention and post-intervention periods were compared to determine the impact of diagnostic stewardship on reducing urine culture orders.

## Results

### Urine cultures

**Figure 2.**
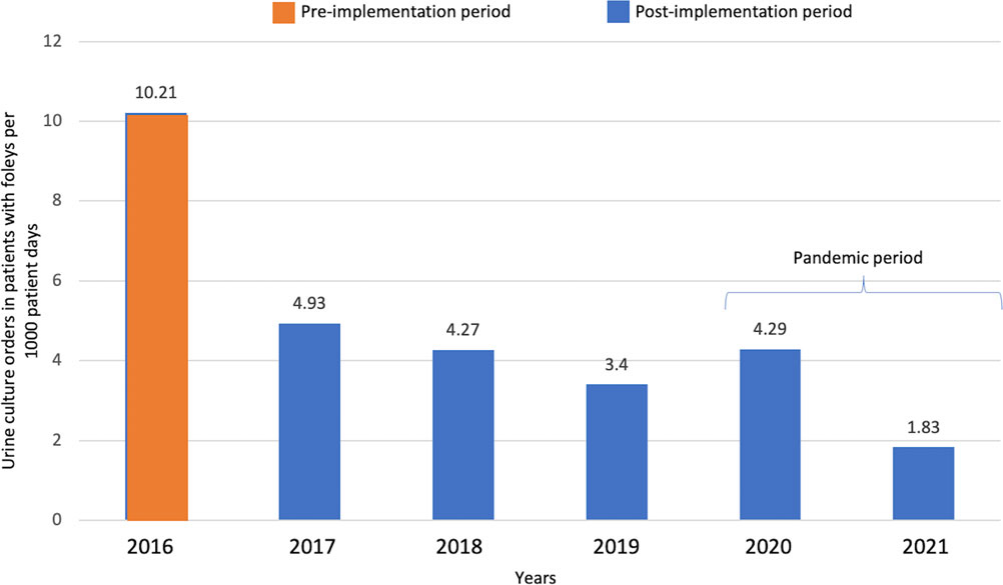
Urine culture orders from urinary catheters per 1000 patient days before and after the diagnostic stewardship intervention.

In the pre-intervention year, 316 urine cultures were ordered, of which 59 were from urinary catheters and 26 were >72 hours from admission (Supplement C). Post-intervention, 222 were ordered in the first year, of which 37 were in patients with urinary catheters and 12 were >72 hours from admission. Percent of cultures from urinary catheters was 27% in the pre-intervention year, followed by 12% in the first post-intervention year, and 17%, 13%, 20%, and 11% subsequently. Urine cultures per 1,000 patient days were 10.21 pre-intervention and 4.93 in the first year after the intervention, a 52% decline (Fig. 2). This was followed by 4.27, 3.40, 4.29, and 1.83.

### CAUTIs

There were 10 CAUTIs pre-intervention, of which 9 occurred in 2014 (4.72 CAUTIs per 1,000 catheter days) and 1 in 2016 (0.7 CAUTIs per 1,000 catheter days). The post-intervention period had no CAUTIs.

### Case audits

Pre-intervention, 19 patients with 23 urine cultures from urinary catheters were reviewed. Among these, one culture (4%) met the IDSA/SCCM criteria (Supplement A). Three cases (16%) met the National Healthcare Safety Network criteria for CAUTIs but did not meet the IDSA/SCCM indications. Post-intervention, 13 cases with 21 urine culture orders underwent prospective audit. Among these, 19 (90%) did not meet the IDSA/SCCM criteria. Average number of days with a urinary catheter was 8. Cases were discussed with ordering providers, and all agreed to cancel orders after feedback. Alternative causes for fever were identified in all cases with canceled orders, including aspiration pneumonitis (4), seizures (1), influenza (2), acute cholecystitis (1), intra-abdominal abscess (2), pneumonia (3), gastroenteritis (3), pancreatitis (1), cerebrovascular accident (1), and tuberculosis (1). Antimicrobials were administered to 78% of cases reviewed in the pre-intervention period and to 53% (7) of patients during the intervention period, a reduction of 32%. Pre-intervention, 54% (12) of cases were in the intensive care unit, compared to 91% (19) of the cases post-intervention.

## Discussion

We successfully implemented a diagnostic stewardship intervention for urine cultures from urinary catheters at a 151-bed safety-net community hospital, which led to a change in urine culture ordering patterns. The percentage of urine cultures from catheters substantially declined from 27% to 12% in the first year, a 52% decline, and urine cultures per 1,000 patient days decreased from 10.21 to 4.93, a 52% decline. This trend continued in subsequent years, underscoring the sustainability of our intervention and its impact. This reduction in urine cultures from urinary catheters represents a true change in the culture of indiscriminate testing. By 2021, only five urine cultures from urinary catheters were ordered. Our intervention was feasible throughout the COVID-19 pandemic and was a key component of our CAUTI prevention strategy.

In the US, over 70% of hospitals have <200 beds and 10% have <25 beds.^8^ Thus, finding effective diagnostic stewardship and CAUTI prevention strategies in these settings is of major importance. Our intervention required no capital expenditures. Therefore, it could be easily replicated in other small hospitals and could be adapted to local infrastructure and staffing. In facilities without ID physicians, antimicrobial stewards or hospitalists can serve as leads for urine culture audit and feedback. No hospital-acquired CAUTIs occurred in our post-intervention period. This holds paramount significance for patient well-being, cost, and hospital quality measures.

Our study had many limitations, including its single-center quasi-experimental design that may limit generalizability. We performed audits only for one year before and after intervention and audited cases from 2014 when our facility had more CAUTIs to compare. Physician staffing changes may contribute to differences in urine culture ordering. Another weakness was our inability to create a report for urine culture orders before 2016 due to changes in our EMR. However, we considered it important to include the COVID-19 pandemic to demonstrate the longitudinal impact of our program.

In conclusion, our diagnostic stewardship initiative was successful at a 151-bed safety-net community hospital. Our findings underscore the role and long-term sustainability of diagnostic stewardship in reducing CAUTIs, enhancing patient care, and promoting evidence-based clinical practices.

## Supporting information

Mena Lora et al. supplementary material Mena Lora et al. supplementary material

## Supplementary material

For supplementary material accompanying this paper visit http://doi.org/10.1017/ash.2024.12. click here to view supplementary material
