# Supplementary material for: Changing the culture: impact of a diagnostic stewardship intervention for urine culture testing and CAUTI prevention in an urban safety-net community hospital
Source: Antimicrob Steward Healthc Epidemiol. 2024 Jan 29;4(1):e14. doi: 10.1017/ash.2024.12 (PMC10897718; doi:10.1017/ash.2024.12)
Supplement: Mena Lora et al. supplementary material [file S2732494X24000123sup001.docx]

**SUPPLEMENT**



Supplement A. Memorandum shared with physicians and nurses.


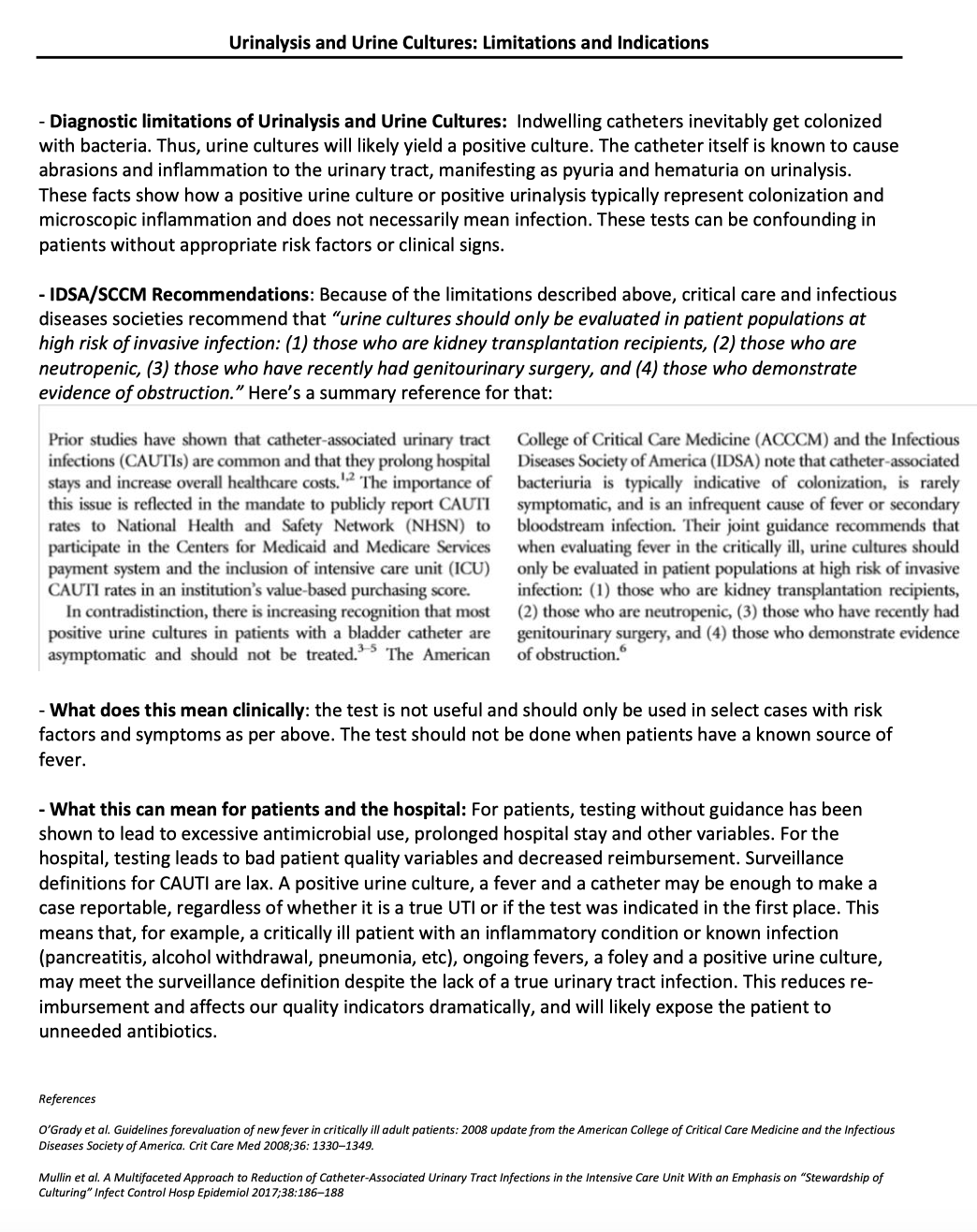

Supplement B. Signage posted in nursing units and areas where urine collection cups were stored.


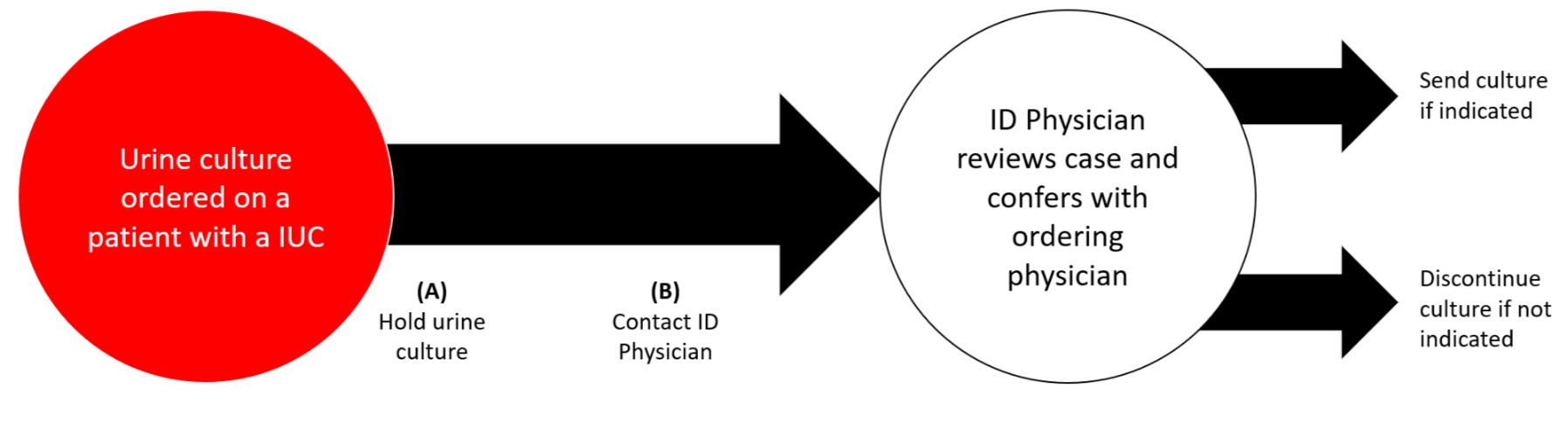


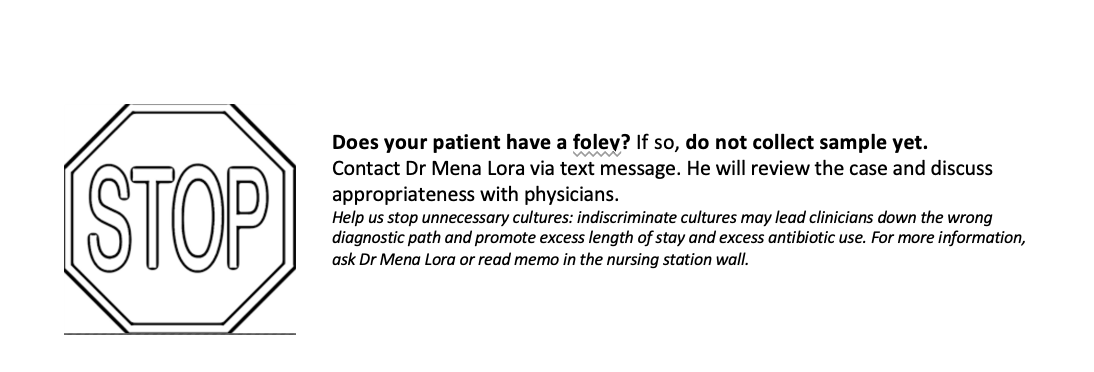


Supplement C. Table with patient days, urine cultures and urine cultures per 1000 patient days for each year before and after our intervention.

| Year | Patient days | Total urine cultures | Urine cultures from urinary catheters | % TOTAL | Urine cultures from urinary catheters collected >72 hours | % urine cultures from urinary catheters | % of IUC form UC both >72h | Urine cultures/patient days x 1000 |
| --- | --- | --- | --- | --- | --- | --- | --- | --- |
| 2016 | 2545 | 215 | 59 | 27% | 26 | 12% | 35% | 10.21611 |
| 2017 | 2431 | 316 | **37** | 12% | **12** | 4% | 32% | 4.93624023 |
| 2018 | 2339 | 222 | 38 | 17% | 10 | 4% | 32% | 4.27533134 |
| 2019 | 2352 | 203 | 28 | 13% | 8 | 4% | 19% | 3.40136054 |
| 2020 | 2791 | 175 | 36 | 20% | 12 | 7% | 40% | 4.29953422 |
| 2021 | 2724 | 158 | 18 | 11% | 5 | 3% | 25% | 1.83553598 |
